# Supplementary material for: Syntactic sugars: crafting a regular expression framework for glycan structures
Source: Bioinform Adv. 2024 Apr 19;4(1):vbae059. doi: 10.1093/bioadv/vbae059 (PMC11069104; doi:10.1093/bioadv/vbae059)
Supplement: vbae059_Supplementary_Data [file vbae059_supplementary_data.docx]

**Syntactic Sugars: Crafting a Regular Expression Framework for Glycan Structures**

Alexander R. Bennett^1^, Daniel Bojar^2,*^

^1^Department of Medical Biochemistry, Institute of Biomedicine, University of Gothenburg, 41390 Gothenburg, Sweden.

^2^Department of Chemistry and Molecular Biology, University of Gothenburg, 41390 Gothenburg, Sweden. Wallenberg Centre for Molecular and Translational Medicine, University of Gothenburg, 41390 Gothenburg, Sweden.

^*^Corresponding author

**Supplementary Figures**


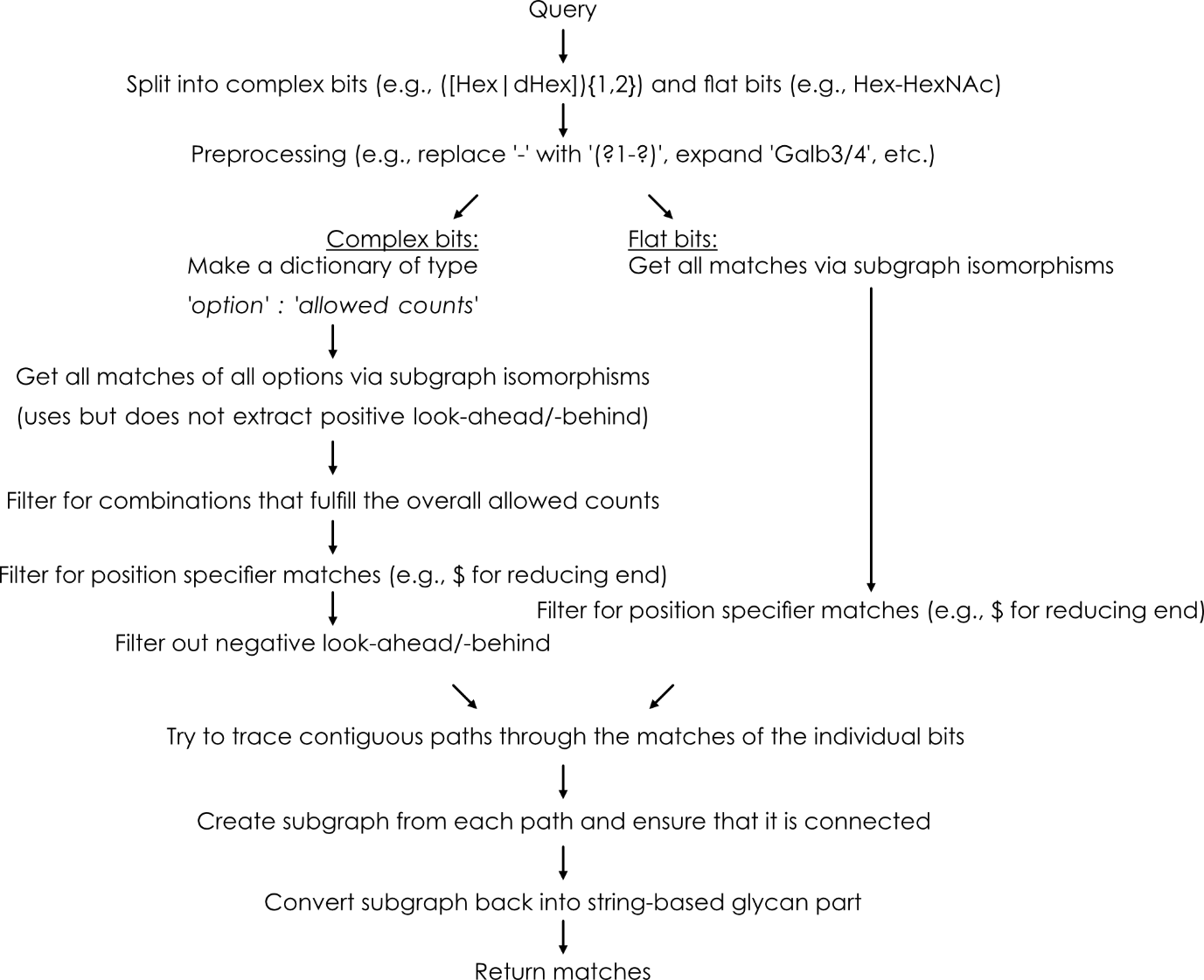


**Supplementary Figure S1. Schematic overview of the developed algorithm for glyco-regular expressions.** Queries are formatted in a blend of typical regular expression notation and IUPAC-condensed formulation of glycans. For simple queries, the *glycowork.motif.regex.motif_to_regex* function can be used to generate properly formatted queries. Further information can be found at <https://bojarlab.github.io/glycowork/motif.html#regex>, in the documentation of glycowork. In general, if a query can be expressed as a single subgraph isomorphism operation, we recommend using *glycowork.motif.graph.subgraph_isomorphism* instead, as it is both more robust and faster. RegEx query execution leverages the subgraph isomorphism functionality that is found, and extensively optimized for performance, in glycowork. Thanks to the universal input functionality of glycowork (automatically detecting and converting nomenclatures), queries can be used on glycans in all major string-based nomenclatures (WURCS, GlycoCT, Oxford, IUPAC-extended, IUPAC-condensed), as well as glycan graphs as networkx objects. Further, glyco-regular expressions can be used with all classes and types of glycans.


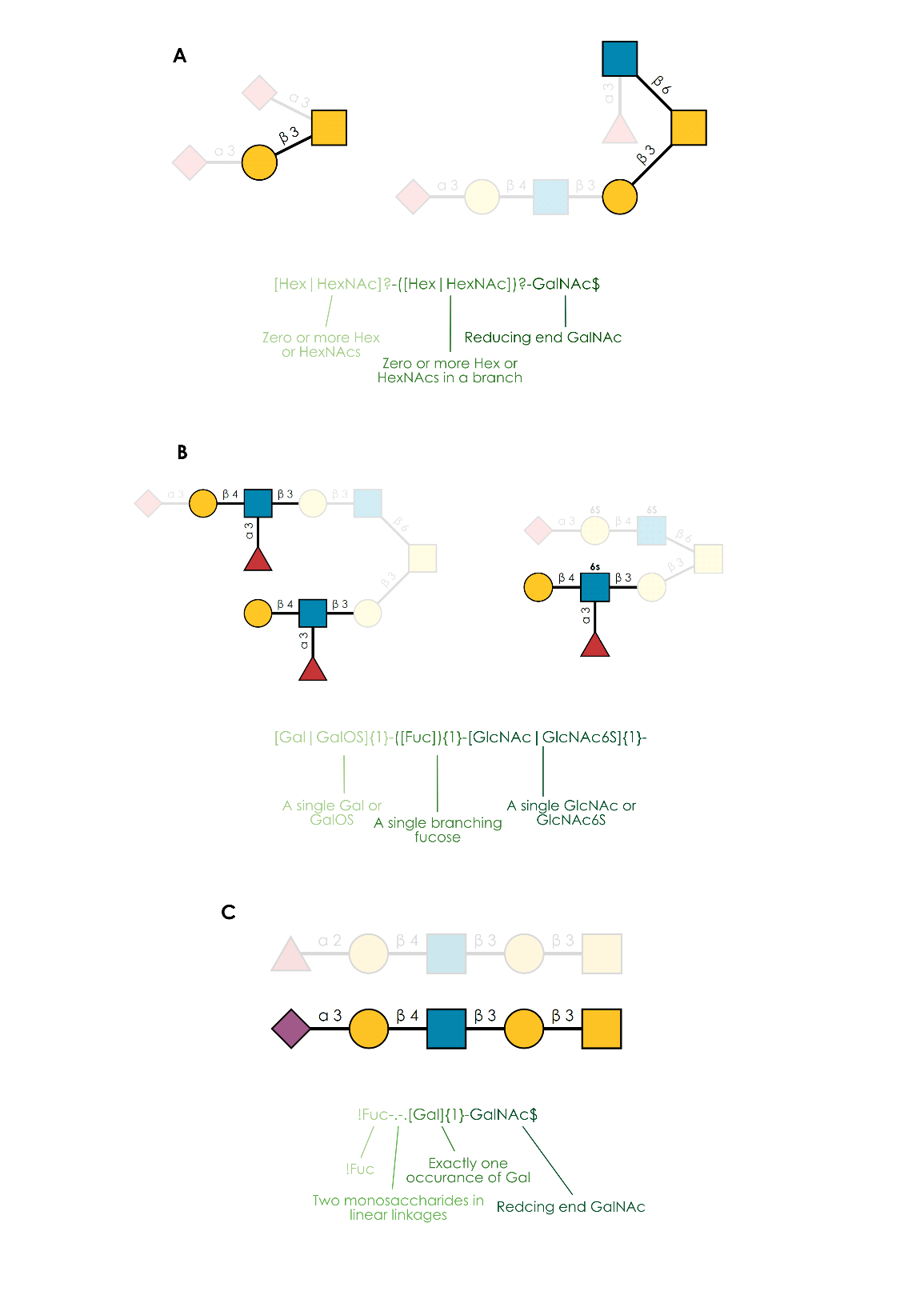


**Supplementary Figure S2. Examples of annotated queries in glyco-regular expressions. A-C)** For three queries, the individual components are described regarding what they are intended to capture. Further, the captured element is highlighted in various SNFG-depicted glycan structures.


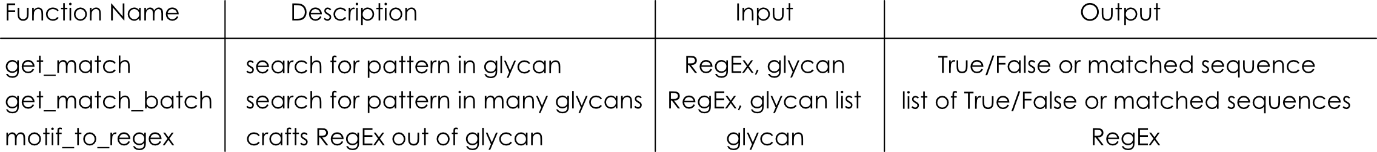


**Supplementary Figure S3. Available functions for end users in *glycowork.motif.regex*.** Depicted are the functions within the *glycowork.motif.regex* module that are intended to be used by end users. The core of our approach is the *get_match* function, which returns either True/False or the matched sequences, depending on the state of the “return_matches” keyword argument. All depicted functions use the universal input system and can be used with glycan sequences in practically any format (WURCS, GlycoCT, Oxford, IUPAC-extended, IUPAC-condensed and variants thereof, glycan graphs), without any preprocessing or format conversion on the side of the user. While the *get_match* function is already present in glycowork v1.1, the *get_match_batch* and *motif_to_regex* functions were only added in glycowork v1.2.
